# RRM2B-Mediated Regulation of Mitochondrial Activity and Inflammation under Oxidative Stress

**DOI:** 10.1155/2015/287345

**Published:** 2015-05-18

**Authors:** Er-Chieh Cho, Mei-Ling Kuo, Jia-hui Cheng, Yu-Chi Cheng, Yi-Chen Hsieh, Yun-Ru Liu, Rong-Hong Hsieh, Yun Yen

**Affiliations:** ^1^Department of Clinical Pharmacy, School of Pharmacy, College of Pharmacy, Taipei Medical University, Taipei 110, Taiwan; ^2^Master Program for Clinical Pharmacogenomics and Pharmacoproteomics, School of Pharmacy, Taipei Medical University, Taipei 110, Taiwan; ^3^Department of Molecular Pharmacology, Beckman Research Institute, City of Hope, Duarte, CA 91010, USA; ^4^School of Nutrition and Health Sciences, College of Public Health and Nutrition, Taipei Medical University, Taipei 110, Taiwan; ^5^Ph.D. Program for Neural Regenerative Medicine, College of Medical Science and Technology, Taipei Medical University, Taipei 110, Taiwan; ^6^Joint Biobank, Office of Human Research, Taipei Medical University, Taipei 110, Taiwan; ^7^Graduate Institute of Cancer Biology and Drug Discovery, College of Medical Science and Technology, Taipei Medical University, Taipei 110, Taiwan

## Abstract

RRM2B is a critical ribonucleotide reductase (RR) subunit that exists as p53-inducible and p53-dependent molecule. The p53-independent regulation of RRM2B has been recently studied, and FOXO3 was identified as a novel regulator of RRM2B. However, the p53-independent regulation of RRM2B, particularly under oxidative stress, remains largely unknown. In this study, we investigated the role of RRM2B underoxidative stress-induced DNA damage and further examined the regulation of mitochondrial and inflammatory genes by RRM2B. Our study is the first to report the critical role of RRM2B in mitochondrial homeostasis and the inflammation signaling pathway in a p53-independent manner. Furthermore, our study provides novel insights into the role of the RR in inflammatory diseases.

## 1. Introduction

Ribonucleotide reductase (RR) catalyzes the conversion of ribonucleoside diphosphates into deoxyribonucleoside diphosphates, playing essential roles in DNA synthesis and repair in humans and influencing vital cellular mechanisms [1–5]. The molecular regulations of the three known RR subunits, RRM1, RRM2, and RRM2B (also called p53R2), have long been studied and reported by our and other groups [6–15]. Previous research has established that RRM2B, a p53-inducible RR subunit, plays vital roles in DNA repair, cell cycle modulation, mitochondrial DNA (mtDNA) synthesis, metastasis suppression, and oxidative stress resistance [1, 6, 7, 9, 16–19]. Mutation or absence of* Rrm2b* in humans results in defective mtDNA, and severe mtDNA depletion has been observed in* Rrm2b*−/− animals [9, 16]. In cells, RRM2B can be regulated by p53 and p73, a p53 family member. In addition, RRM2B regulates the p53-dependent cell cycle for DNA damage [6]. RRM2B can suppress the metastasis and proliferation of different cancer cells [20]. A study on a* Rrm2b*-knockout animal model indicated that the intactness of the RRM2B subunit is critical for maintaining chromosomal stability and that the loss of RRM2B results in plasmacytic neoplasms [21]. Furthermore, RRM2B expression is correlated with improved survival in some cancers, whereas a more progressive phenotype of certain other cancers complicates the RRM2B regulatory pathway [22–24].

Several reports have suggested that RRM2B regulates critical cellular mechanisms irrespective of the p53 status. First, although the p53-mediated RRM2B induction is inhibited in the p53-deficient mouse embryonic fibroblasts cells, it has been observed that RRM2B is expressed at basal levels [16]. Furthermore, a high RRM2B expression has been observed in various p53-deficient cancer cells, and it continues to influence mitochondrial functions irrespective of the p53 status, suggesting that RRM2B-mediated mitochondrial homeostasis is independent of functional p53, and other factors are involved in RRM2B regulation [25, 26]. The findings of our recent study are consistent with those of the aforementioned studies, in which the tumor suppressor FOXO3 binds to the RRM2B promoter and activates RRM2B transcription in a p53-independent manner under physiological conditions [27].

RRM2B is a unique member among the RR subunits that can resist reactive oxygen species (ROS) [8, 19, 25, 28, 29]. However, little is known about the detailed mechanisms and the functional regulations of RRM2B under oxidative stress. We studied the functional regulations of RRM2B in cancer cells under oxidative stress and observed that RRM2B plays a crucial role in the regulation of mitochondrial and inflammation pathways in a p53-independent manner.

## 2. Materials and Methods

### 2.1. Cell Lines, Plasmids, and Stable Cell Line Production

Cells from ATCC were cultured in DMEM medium containing fetal bovine serum (10%) and penicillin/streptomycin (1%) incubated at 37°C with 5% CO_2_. RRM2B overexpression and shRNA plasmids were described before [28]. Stable cell lines were established by infection and selection as described [28].

### 2.2. Immunofluorescence

The assay was performed as described [30]. In brief, H1299 cells were seeded on coverslips, fixed, permeabilized, stained with primary antibodies, anti-RRM2B (Rockland) and *γ*-H2AX (Active Motif), and secondary antibodies (Invitrogen), washed, and then mounted with DAPI (Invitrogen) onto the slides. Samples were analyzed by immunofluorescence microscope system, and the scale bars indicate 10 *μ*M.

### 2.3. Western Blot Analysis and Cytoplasmic/Nuclear Protein Extraction

Cell extracts were prepared and analyzed as described before [31]. Antibodies used in this assay were RRM2B (Rockland), *γ*-H2AX (Active Motif), GAPDH (Santa Cruz and GeneTex), VDAC1, COX4, p-NF*κ*B, p-p38, and p-I*κ*B (GeneTex). The cytoplasmic/nuclear extraction kit was applied to separate cytoplasmic and nuclear fractions as described in the manufactory protocol (TOOLS) before processing to the western on phospho antibodies. Quantitative protein expression relative to GAPDH was analyzed by Image J where applicable.

### 2.4. Mitochondrial Mass Measurement

Cells were treated with H_2_O_2_, harvested, washed with PBS, and stained with MitoTracker Green probes (Invitrogen) of 50 nM in serum-free medium for 30 mins. Cells were washed with PBS and analyzed by FACSCanto II cytometer. Fluorescent intensity was analyzed by FACSCanto II program.

### 2.5. Real-Time PCR Analysis

Total RNA was isolated by Trizol, and then RNA was reverse transcribed (Quanta) to obtain cDNA for PCR [32]. cDNA was subjected to real-time PCR using the SYBR Green PCR reagents kit (Stratagene) as described [33]. For quantitation of mtDNA copy number,* Nd1* gene level was quantified by q-PCR and normalized to *β*-*Actin*. Due to the space limitation, primers are provided upon request.

### 2.6. Statistical Methods

The statistical analysis was done as described [27]. In brief, Student's *t*-test was used for *P* value calculation, and the star ^∗^ stands for *P* < 0.05. The results shown in this paper are representative data.

## 3. Results

### 3.1. Impact of RRM2B on H_2_O_2_-Mediated DNA Damage Resistance in p53-Deficient Cells

**Figure 1:**
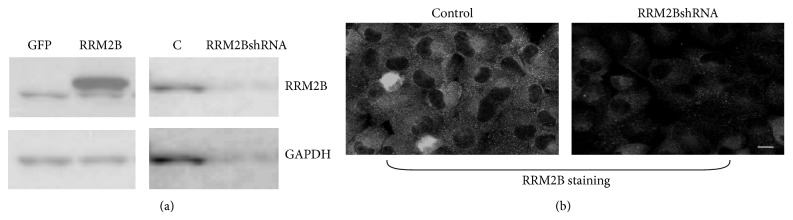
Expression of RRM2B in the H1299 stable cell lines. H1299 stable cell lines were selected as described in the Section 2. Cells were harvested for (a) Western blot analysis or (b) fixed for immunofluorescence assay. Anti-RRM2B and GAPDH were applied in the assays.

Although it is a known fact that RRM2B can resist ROS, little is known about the detailed regulatory mechanisms. We hypothesized that the oxidative resistance of RRM2B is independent of p53. Stable H1299 cell lines without the functional p53 protein containing overexpressed RRM2B or RRM2BshRNA and their respective controls were included in this study, as described previously, [27] and RRM2B expression was evaluated using Western blot analysis (Figure 1(a)) and an immunofluorescence assay (Figure 1(b)).

**Figure 2:**
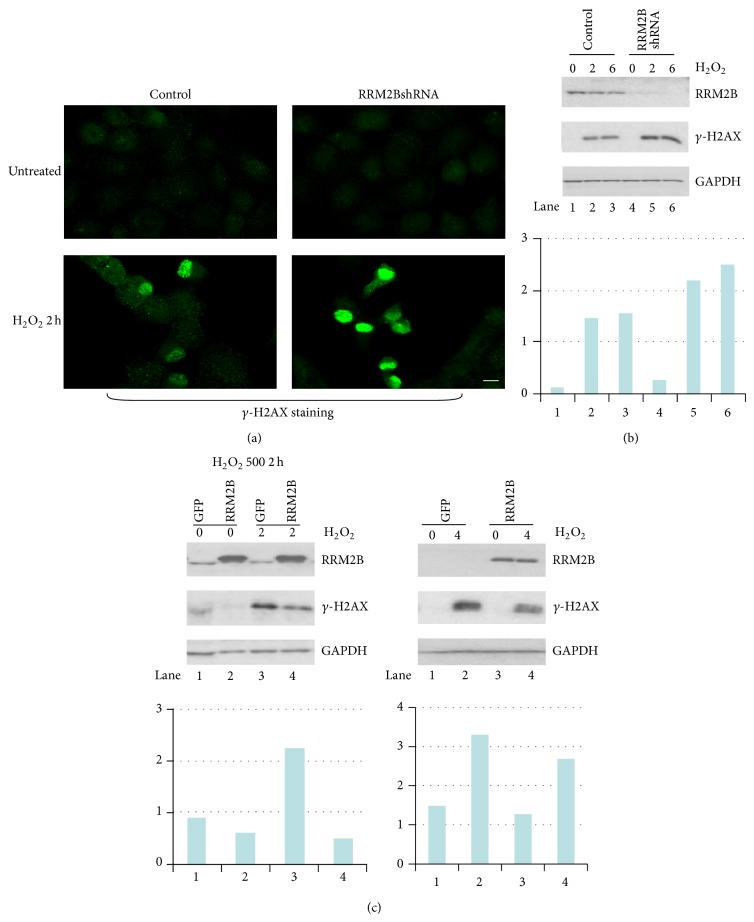
RRM2B functions in H_2_O_2_-mediated DNA damage resistance in p53-deficient cells. (a) H1299 stable cells were treated with 500 *μ*M H_2_O_2_ for 2 hours where applicable for immunofluorescence assay analysis with anti-*γ*-H2AX antibody. (b)-(c) Two pairs of cell lines expressing control vectors, RRM2BshRNA and RRM2B expressing vector, were treated with H_2_O_2_ and harvested at different time points for Western blots analysis with indicated antibodies. (*n* = 2) Image J was used to normalize *γ*-H2AX expression to GAPDH.

For investigating RRM2B regulation under oxidative stress, hydrogen peroxide (H_2_O_2_), a type of oxidative stress-inducing ROS, was used as the source of oxidative stress in this study. Cells were treated with H_2_O_2_ for 2 hours and fixed for immunofluorescence staining, in which *γ*-H2AX foci induction indicated DNA damage. The results suggested that stronger *γ*-H2AX signals were induced by cells expressing RRM2BshRNA than by the control cells (Figure 2(a)), suggesting that greater DNA damage was associated with lower RRM2B expression in cells.

For the Western blot analysis, we performed the experiment at different time intervals, and the *γ*-H2AX signals on the blots were quantified relative to the GAPDH activity for quantitative estimation and analysis. Consistent RRM2B depletion was observed, which was correlated with the induction of *γ*-H2AX signals under H_2_O_2_ treatment (Figure 2(b)). Similarly, we observed low *γ*-H2AX foci signals under H_2_O_2_ treatment in RRM2B overexpressed cells (Figure 2(c)). In summary, the results suggested that RRM2B plays a protective role in securing cells against oxidative stress-induced DNA damage in a p53-independent manner.

### 3.2. RRM2B-Mediated Impact on Mitochondrial Regulation under Oxidative Stress

**Figure 3:**
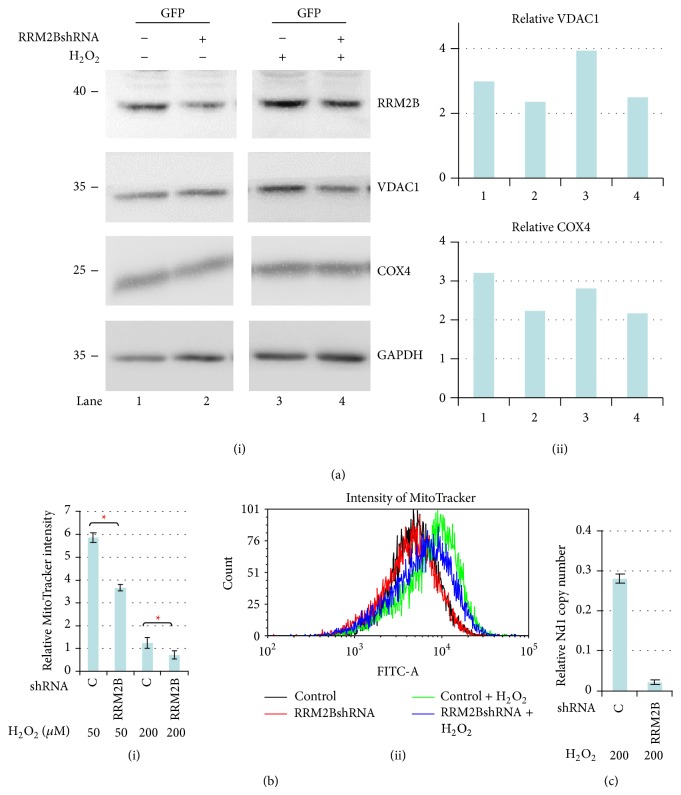
RRM2B impacts on mitochondrial homeostasis through regulating mitochondrial genes and copy number under oxidative stress. (a) Cells were treated with H_2_O_2_ and harvested for Western blot analysis using RRM2B and mitochondrial proteins VDAC1 and COX4 antibodies. GAPDH served as control. Image J was used to normalize VDAC1 and COX4 expression relative to GAPDH, and the normalized figures were shown in 3(a)(ii). (b)(i) H1299 stable cells treated with indicated concentrations of H_2_O_2_ were harvested 24 hours later for FACS analysis. The detection of MitoTracker was described in Section 2. The intensity of the MitoTracker Green signal relative to untreated control cells is shown here (means ± SEM, *n* = 2). (b)(ii) Cells were treated with H_2_O_2_ and harvested at 24-hour time point and underwent FACS analysis. MitoTracker Green probe was used for cell staining, and this figure shows the MitoTracker intensity of the representative data from (b)(i). (c) Cells were treated with 200 *μ*M H_2_O_2_ and harvested 24 hours later for Q-PCR analysis. Expression of normalized* Nd1* relative to untreated control cells is shown (means ± SEM, *n* = 2).

Next, we examined the role of RRM2B in regulating mitochondrial proteins under oxidative stress in p53-deficient H1299 cells. Only stable cells expressing RRM2BshRNA or those acting as control that were subjected to or excluded from H_2_O_2_ treatment were included in this study. The cells were then harvested for Western blot analysis, in which the expression of mitochondrial proteins, voltage-dependent anion channel 1 (VDAC1), and cytochrome c oxidase subunit IV (COX4) [34–36] were examined along with RRM2B expression and GAPDH activity (Figure 3(a)(i)). In addition, the relative expression levels of VDAC1 and COX4 were analyzed and are shown in Figure 3(a)(ii). The results suggested that the expression levels of both proteins, particularly VDAC1, were more pronounced under H_2_O_2_ treatment, and the expression levels decreased on RRM2B downregulation, confirming that RRM2B regulated the mitochondrial content (Figure 3(a)).

To further understand the impact of RRM2B on the mitochondria, the mitochondrial mass was measured. FACS was performed using a MitoTracker Green probe for detecting the mitochondrial mass in stable H1299 cells under H_2_O_2_ treatment. Under oxidative stress, a significant decrease was observed in the relative mitochondrial mass of the cells expressing RRM2BshRNA, indicating the protective role of RRM2B in mitochondrial homeostasis (Figure 3(b)(i)), which is in agreement with our previous finding [25]. The representative examples of the intensity of MitoTracker on FACS are shown in Figure 3(b)(ii).

Moreover, quantitative PCR was used for measuring the relative mtDNA copy number. The relative copy number of the NADH dehydrogenase subunit 1 (*Nd1*), a mitochondrial gene [37], under H_2_O_2_ treatment was measured using quantitative PCR. Low RRM2B expression resulted in a low* Nd1* copy number (Figure 3(c)), suggesting that, under oxidative stress, RRM2B exerts protective effects on the mtDNA content.

The results establish that the RRM2B pathway affects mitochondrial homeostasis under oxidative stress, which is independent of the action of functional p53.

### 3.3. RRM2B Affects the Inflammation Pathway under Oxidative Stress

**Figure 4:**
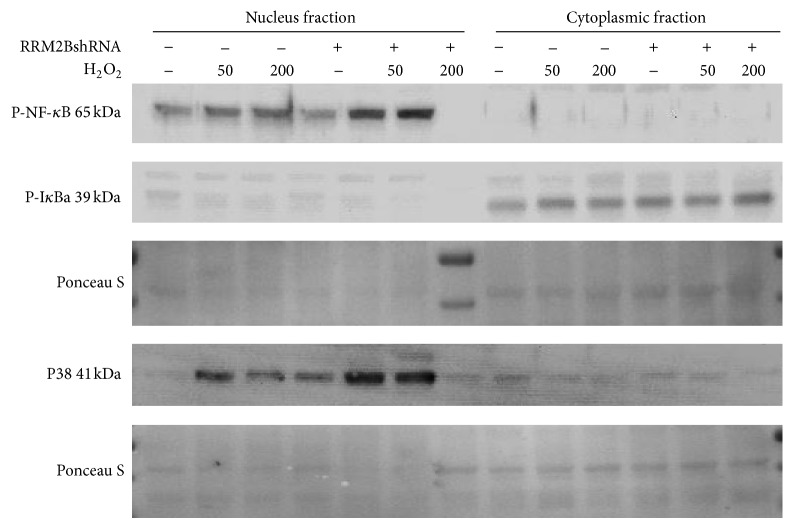
RRM2B regulates inflammatory signaling pathway under oxidative stress. (a) Stable cells were treated with 50 or 200 *μ*M H_2_O_2_ and harvested 2 hours later, and the lysates were separated into nuclear and cytoplasmic fractions for Western blot analysis. Antibodies against phosphorylated NF-*κ*B, phosphorylated I*κ*B, and phosphorylated p38 were applied, and GAPDH was used as loading control.

Oxidative stress mediated by H_2_O_2_ triggers the inflammation pathway [38, 39], and human inflammatory diseases have long been associated with NF-*κ*B or p38 signaling or both [40–42]. However, the role of RRM2B in the regulation of inflammation has not been investigated. NF-*κ*B and p38 are complex pathways regulating various cellular mechanisms [41, 43–46]. The NF-*κ*B pathway can be activated by various proinflammatory cytokines and is therefore considered a proinflammatory signaling pathway. In addition, extracellular stimuli such as UV light, growth factors, and inflammatory cytokines result in p38 activation. In this study, we investigated the functional role of RRM2B in oxidative stress-mediated NF-*κ*B and p38 signaling. Stable H1299 control or RRM2BshRNA cells were treated with H_2_O_2_ and harvested 2 hours later, and the lysates were separated into cytoplasmic and nucleolus fractions. Western blot analysis was performed using phosphorylated NF-*κ*B, phosphorylated I*κ*B, and phosphorylated p38 antibodies for identifying NF-*κ*B and p38 activation in cells (Figure 4).

The results showed that nuclear NF-*κ*B and p38 signaling on H_2_O_2_ treatments were more pronounced in the cells expressing RRM2BshRNA than in the control cells (Figure 4), suggesting that a stronger inflammation signal was induced by RRM2B depletion.

**Figure 5:**
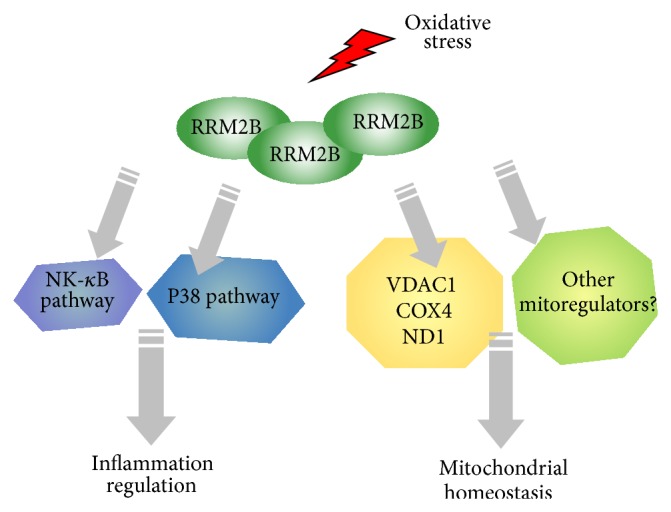
A model of RRM2B-mediated regulation of inflammatory NF-*κ*B and p38 pathways and mitochondrial pathways upon oxidative stress.

In summary, the findings suggest for the first time that RRM2B can functionally regulate the mitochondrial and inflammatory pathway and that these RRM2B-mediated regulations are independent of p53.  Figure 5 illustrates a model summarizing the current findings of RRM2B regulation, suggesting that, under oxidative stress, RRM2B plays critical roles in the upregulation of genes involved in the mitochondrial and inflammation pathways.

## 4. Conclusion and Discussion

RRM2B is a unique member of the RR enzyme that exhibits anti-ROS potential. It was demonstrated that RRM2B suppressed ROS activation mediated by oxidative stress and is highly induced in a p53-dependent manner during senescence [28]. In our recent study, FOXO3 was observed to be a novel regulator of RRM2B [27]. In this study, the critical role of RR2MB in regulation of mitochondrial and inflammation pathways under oxidative stress in a p53-independent manner was reported for the first time.

RRM2B plays critical roles in vital cellular mechanisms such as DNA replication, and low RRM2B expression sensitizes cancer cells under various stresses, and therefore studies have suggested that low RRM2B expression can potentially be considered a chemosensitizer for cancer treatment [2, 47, 48]. In this study, H1299 cells were subjected to oxidative stress, and the induced *γ*H2AX foci signals were stronger in RRM2BshRNA cells, indicating that the chemosensitivity may generate equal outcomes in p53-deficient cancer cells. Additional therapeutical applications remain to be uncovered.

As mentioned,* RRM2B* mutation results in severe mtDNA depletion [9]. In this study, we further demonstrated that the presence of RRM2B affects mitochondrial protein expression. Mitochondrial mass was damaged under low RRM2B expression and was further destroyed under oxidative stress. Findings of previous studies and the data in the present study indicate that the intactness of RRM2B is critical for complete functioning of the mitochondria, despite the presence of functional p53.

Oxidative stress has been shown to have a strong association with inflammation pathways. Moreover, our previous study using RRM2B-knockout animal models suggested that RRM2B is critical in maintaining chromosomal stability and preventing chronic inflammation-associated tumorigenesis [21]. In this study, we demonstrated that the NF-*κ*B and p38 signaling pathways were upregulated by oxidative stress, particularly under low RRM2B conditions, which is in agreement with our previous finding, suggesting that RRM2B is crucial in preventing chronic inflammation and acts by inhibiting the NF-*κ*B and p38 pathways.

Both NF-*κ*B and p38 signaling pathways affect vital cellular regulatory mechanisms that include inflammation and apoptosis [46, 49]. Therefore, accompany with our data, the RRM2BshRNA cells could potentially trigger stronger inflammation and apoptosis signals under DNA damage conditions. Recently, more complex roles of NF-*κ*B have been suggested in NF-*κ*B activation in pro- and anti-inflammation processes and pro- and antiapoptosis [49, 50], which may depend on the nature of the model systems. The status of p53 further increased the complexity. Our current study provides new insights into the role of RR in inflammatory diseases, and the intriguing regulation mechanisms of RRM2B under oxidative stress in inflammation remain to be explored.
